# Effect of Garlic Extract on the Erythrocyte as a Simple Model Cell

**DOI:** 10.3390/ijms25105115

**Published:** 2024-05-08

**Authors:** Paulina Furdak, Grzegorz Bartosz, Ireneusz Stefaniuk, Bogumił Cieniek, Edyta Bieszczad-Bedrejczuk, Mirosław Soszyński, Izabela Sadowska-Bartosz

**Affiliations:** 1 Laboratory of Analytical Biochemistry, Institute of Food Technology and Nutrition, College of Natural Sciences, Rzeszow University, 4 Zelwerowicza Street, 35-601 Rzeszow, Poland; paulinaf2@o2.pl (P.F.); gbartosz@ur.edu.pl (G.B.); ebieszczad@ur.edu.pl (E.B.-B.); 2 Doctoral School, University of Rzeszow, 16C Rejtana Street, 35-959 Rzeszów, Poland; 3 Institute of Materials Engineering, College of Natural Sciences, University of Rzeszow, 1 Pigonia Street, 35-310 Rzeszow, Poland; istefaniuk@ur.edu.pl (I.S.); bcieniek@ur.edu.pl (B.C.); 4 Department of Oncobiology and Epigenetics, Faculty of Biology and Environmental Protection, University of Lodz, Pomorska 141/143, 90-236 Lodz, Poland; miroslaw.soszynski@biol.uni.lodz.pl

**Keywords:** erythrocyte, garlic, thiol, glutathione, hemoglobin, Heinz bodies, reactive oxygen species, lipid peroxidation

## Abstract

Garlic is known to have diverse effects on mammalian cells, being cytotoxic, especially to cancer cells, but also protect against oxidative stress. Mammalian erythrocyte is a simple cell devoid of intracellular organelles, protein synthesis ability, and most signaling pathways. Therefore, examination of the effects of garlic on erythrocytes allows for revealing primary events in the cellular action of garlic extract. In this study, human erythrocytes or erythrocyte membranes were exposed to garlic extract at various dilutions. Hemoglobin oxidation to methemoglobin, increased binding of hemoglobin to the membrane, and formation of Heinz bodies were observed. Garlic extract depleted acid-soluble thiols, especially glutathione, and induced a prooxidative shift in the cellular glutathione redox potential. The extract increased the osmotic fragility of erythrocytes, induced hemolysis, and inhibited hemolysis in isotonic ammonium chloride, indicative of decreased membrane permeability for Cl^−^ and increased the membrane fluidity. Fluorescent probes indicated an increased level of reactive oxygen species and induction of lipid peroxidation, but these results should be interpreted with care since the extract alone induced oxidation of the probes (dichlorodihydrofluorescein diacetate and BODIPY C11). These results demonstrate that garlic extract induces oxidative changes in the erythrocyte, first of all, thiol and hemoglobin oxidation.

## 1. Introduction

Garlic (*Allium sativum* L.) is one of the most commonly used food components, not only as a spice for taste fortification but, first of all, for its rich pro-health properties [1,2,3]. Two main classes of garlic compounds, responsible for the biological effects of garlic, are organosulfur compounds (OSCs) and phenolics. The antibacterial [4], antiviral [5], anticancer [6], and anti-inflammatory [7] effects of garlic have been attributed chiefly to OSCs while antioxidant capacity of garlic is mainly dependent on phenolics [8,9] and vitamin C [10,11]. OSCs, on the other hand, exert a prooxidant action, reacting with low molecular-weight thiols as well as inducing oxidative stress, although they may also exert antioxidant action in cells [12,13,14].

The multicomponent system of antioxidant and prooxidant compounds present in garlic can be expected to exert complex action on cells. While numerous in vitro studies have mainly demonstrated cytotoxic activity in garlic, the in vivo cellular effects are more difficult to assess. Erythrocytes, among other cells, have also been studied in this respect and diverse effects, both beneficial and adverse, were reported, apparently dependent on the dose, method of administration, and co-treatment.

Rats given garlic extract 5 g/kg body weight (bw) daily intraperitoneally for 30 days developed anemia and had higher malondialdehyde (MDA) levels, catalase and superoxide dismutase (SOD) activities, and protein carbonyl and free iron levels in erythrocytes [15]. Consumption of garlic (150 mg pernumber kg bw every other day for three weeks) by rats resulted in a decrease in erythrocyte count, an increase in the level of reticulocytes, and fragmented erythrocytes and schistocytes [16]. High garlic intake (4% in the diet) was reported to induce hemolytic anemia in rats [17]. Nevertheless, rats receiving a lower dose of garlic (garlic extract, 500 mg per kg body weight for 1 month) had increased erythrocyte counts [18]. In dogs given garlic extract (5 g garlic/kg bw daily for 7 days), erythrocyte count and Hct were decreased to a minimum value on days 9 to 11. Heinz body formation, an increase in reduced glutathione (GSH) concentration, and the number of leccentrocytes were detected in these dogs. However, no dog developed hemolytic anemia [19].

In rats fed a high-fat diet, inclusion of 2% garlic extract into the diet attenuated the loss of glutathione (GSH) and total thiols, increase in the MDA level and glutathione peroxidase (GPx) activity, and ameliorated changes in the erythrocyte osmotic fragility, Ca^2+^-Mg^2+^-ATPase activity of erythrocytes, and oxidative stress induced by the fat-rich diet, but nonetheless increased the level of methemoglobin [20,21]. In rabbits fed oxidized rapeseed oil for up to 24 months, the inclusion of garlic (1%) in the diet prevented a decrease in SOD activity and increase in MDA content, and increased GPx activity [22]. In mice exposed to lead acetate, administration of garlic extract prevented a decrease in the erythrocyte count, attenuated the increases in erythrocyte reactive oxygen species (ROS) level and SOD activity, and a decrease in catalase activity; it also restored erythrocyte lifespan and flippase and scramblase activities and prevented decreases in the GSH/GSSG and NADPH/NADP ratios [23]. Raw garlic administered in food (250 mg/kg bw) to rats protected against nickel intoxication effects on erythrocytes including a decrease in erythrocyte count, increase in MDA and GSH levels, and increase in SOD, catalase, and GPx activities [24]. Garlic consumption by elderly subjects (0.1 g per kg bw for 1 month) was reported to reduce erythrocyte MDA level and increase activities of SOD, catalase, and GPx [25]. In patients with sickle cell anemia ingesting garlic extract decreased the content of Heinz bodies; this extract was suggested to be a potential therapeutic agent to ameliorate complications of sickle-cell anemia [26]. On the other hand, four-week administration of garlic and onion extracts to rats (150 mg/kg bw) induced a decrease in the osmotic fragility of erythrocyte [27].

These data prompted us to examine the direct effects of garlic on erythrocytes in vitro. Erythrocytes are simple cells devoid of mitochondria and lacking the possibility of protein synthesis, easily accessible in high amounts from blood. Their use can allow for detecting initial garlic-induced changes, which can be hidden and counteracted by adaptive reactions in more complex cells. Several such studies have been performed (see Section 3) but were usually limited to a single or a few parameter(s); this study aimed to obtain a more comprehensive picture of garlic-induced changes. We used extracts of Spanish garlic, proved to be the most cytotoxic to ovarian cancer cells from among several garlic preparations tested [28].

## 2. Results

**Figure 1:**
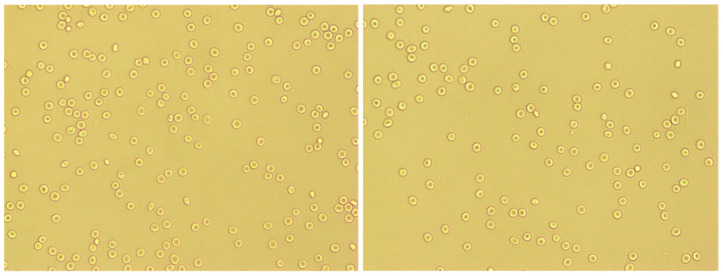
Phase contrast microphotographs of control erythrocytes (**left**) and erythrocytes incubated with 50% garlic extract (**right**). Magnification: 400×.

**Figure 2:**
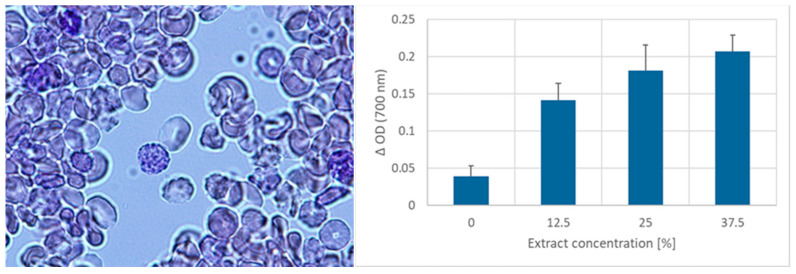
Heinz bodies are formed in erythrocytes incubated with the garlic extract. (**Left**) Erythrocytes were incubated with 50% garlic extract for 1 h at 37 °C and stained supravitally with methyl violet. Magnification: 1000×. (**Right**) Difference in the optical density of hemolysates before and after centrifugation as a measure of the content of Heinz bodies.

Incubation (1 h) of erythrocytes in the presence of garlic extract in PBS did not provoke significant morphological changes in the cells (Figure 1) but induced the formation of Heinz bodies. The formation of Heinz bodies was confirmed by turbidimetric measurements (Figure 2).

**Figure 3:**
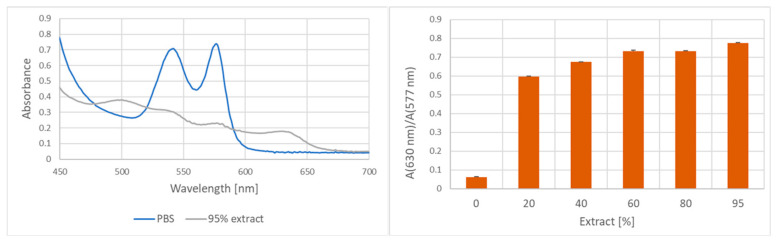
Oxidation of hemoglobin by the garlic extract. (**Left**) Absorption spectra of the control hemolysate and hemolysate of erythrocytes incubated with 95% garlic extract. (**Right**) Dependence of hemoglobin oxidation on the concentration of the garlic extract.

The visible effect of incubation of erythrocytes with the garlic extract was the oxidation of hemoglobin to methemoglobin causing a change in the color of the erythrocyte suspensions from red to red-brown. This was reflected by changes in the absorption spectra of the hemolysates. The extent of hemoglobin oxidation, measured as the ratio of absorbances at 630 nm (absorption maximum of methemoglobin) and 577 nm (absorption maximum of oxyhemoglobin), increased with increasing concentration of the extract (Figure 3).

Incubation with the garlic extract increased the osmotic fragility of erythrocytes: while the NaCl concentration causing 50% hemolysis of control erythrocytes (c_50_) was 63.8 ± 0.2 mM, the c_50_ for erythrocytes incubated with 50% garlic extract was 65.9 ± 0.3 mM (*p* < 0.001). 

**Figure 4:**
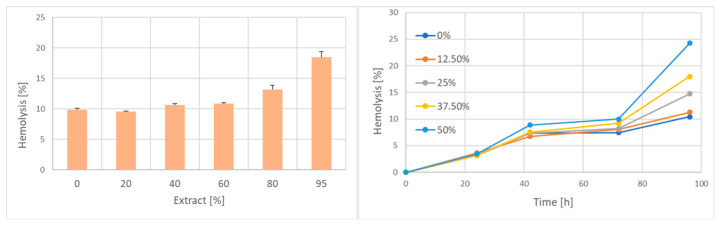
Hemolysis induced by incubation of erythrocytes with the garlic extract. (**Left**) immediate hemolysis; (**Right**) time course of hemolysis during post-exposure incubation at 4 °C.

Incubation of erythrocytes with the extract induced slight hemolysis, depending on the concentration of the extract (Figure 4, left). When erythrocytes exposed to the garlic extract were washed and incubated at 4 °C with intermittent mixing, dose-dependent induction of delayed hemolysis was observed, indicative of garlic-induced damage to membrane structure and/or the ionic equilibrium of the cells (Figure 4, right).

**Figure 5:**
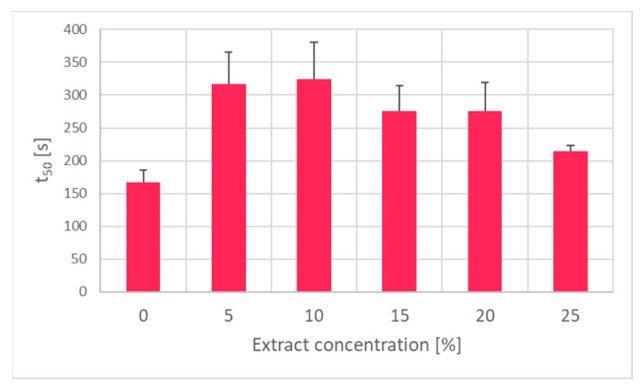
Effect of incubation with the garlic extract on the time required to achieve 50% hemolysis of erythrocytes in 150 mM solution of ammonium chloride.

Hemolysis in isotonic ammonium chloride solution, which is a measure of membrane permeability to chloride anions, was inhibited by incubation of erythrocytes with the garlic extract (Figure 5).

**Figure 6:**
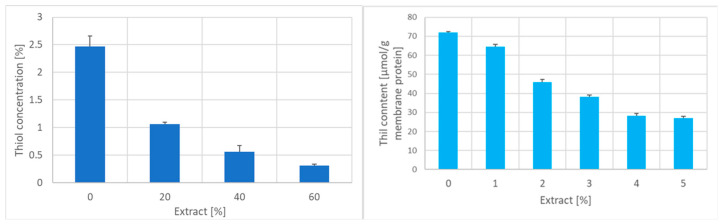
Effect of incubation with the garlic extract on the concentration of erythrocyte acid-soluble thiol groups (**left**) and the membrane content of thiol groups (**right**).

Incubation of erythrocytes with the garlic extract decreased the level of intracellular acid-soluble thiol groups (Figure 6, left) and, even more dramatically, of intracellular reduced glutathione (GSH) and oxidized (GSSG) glutathione. Although the GSH/GSSG ratio decreased in erythrocytes treated with garlic extract, indicating an increase in the extent of oxidation of glutathione, it is obvious from the comparison of magnitudes of concentration changes of both forms of glutathione and simultaneously that most of the GSH is lost in a process different from oxidation to GSSG. The redox potential of the remaining glutathione increased in erythrocytes incubated with the garlic extract, the prooxidative shift reaching up to +90 mV (Table 1).

**Figure 7:**
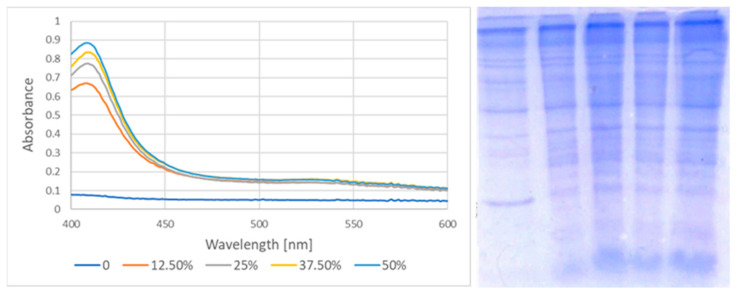
Binding of hemoglobin to membranes in erythrocytes incubated with various dilutions of the garlic extract. (**Left**) Absorption spectra of erythrocyte membranes dissolved in 1% SDS. Concentration of the extract. (**Right**) SDS-PAGE electropherograms of membrane proteins of erythrocytes incubated with the garlic extract. Extract concentrations (from left to right): 0%, 12.5%, 25%, 37.5%, and 50%.

Incubation of erythrocytes with the garlic extract induced membrane binding of hemoglobin demonstrated by both increased absorbance of hemoglobin at the Soret band in the membrane preparations and as the increased percentage of hemoglobin in the densitometric scans of membrane proteins separated by SDS-PAGE (Figure 7, Table 2).

**Figure 8:**
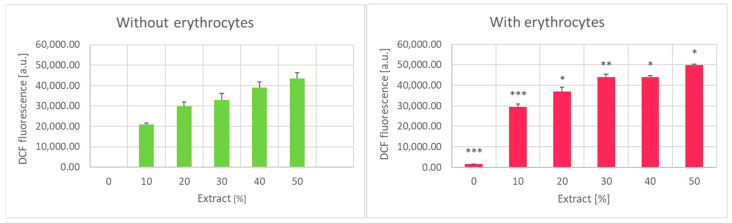
Effect of garlic extract on the level the oxidation of H_2_DCF-DA in the absence (**left**) and in the presence of erythrocytes (**right**). * *p* < 0.05, ** *p* < 0.01, *** *p* < 0.001 (DCF fluorescence in the presence of erythrocytes vs. in the absence of erythrocytes).

Garlic extract appeared to increase the level of intracellular reactive oxygen species (ROS) in erythrocytes detectable by oxidation of 2′,7′-dichlodihydrofluorescein diacetate (H_2_DCF-DA). However, garlic extract itself induced significant oxidation of the probe, which can put in question the validity of this assay as applied to erythrocytes. Nevertheless, the H_2_DCF-DA oxidation to dichlorofluorescein (DCF) in the presence of erythrocytes was always significantly higher than in the absence of erythrocytes, indicating that the extract indeed induces ROS formation in erythrocytes (Figure 8).

**Figure 9:**
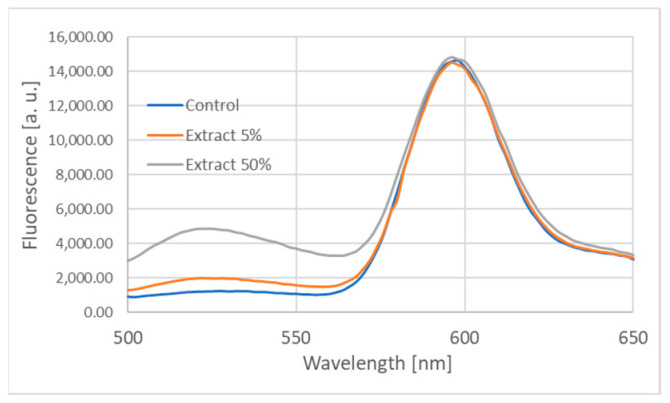
Fluorescence spectra of BODIPY C11 in control erythrocyte membranes and membranes incubated with garlic extract.

Incubation with garlic extract seemed to induce peroxidation of erythrocyte membrane lipids monitored as the increase in the ratio of green to red fluorescence peaks (in our measurements the ratio of fluorescence intensity at 526 and 598 nm) as estimated with the BODIPY C11 probe (Figure 9, Table 3). However, an analogous increase was observed upon incubation of the probe with the garlic extract alone, in the absence of erythrocyte membranes. Therefore, this method of assessment of membrane lipid peroxidation is not reliable in the presence of garlic extract. An independent method of assessment of membrane lipid peroxidation, employing the reaction of lipid peroxidation products with thiobarbituric acid, did not show any lipid peroxidation in erythrocyte membranes incubated with the garlic extract. Likewise, the garlic extract did not induce any protein carbonylation detectable with DNPH.

Incubation of erythrocyte membranes with the garlic extract resulted in an increase in membrane fluidity estimated with pyrene (Table 4) and with the spin probes 5-DS and 16-DS (Table 5).

Garlic extract at the concentration of 50% caused the total (16-DS) or partial (5-NS) disappearance of the EPR signal of the probes, precluding the possibility of reliable estimation of membrane fluidity. Reduction of a water-soluble spin probe 2,6,6,6-tetramethylpiperidine-1-oxyl (TEMPOL) by the garlic extract was also observed.

**Figure 10:**
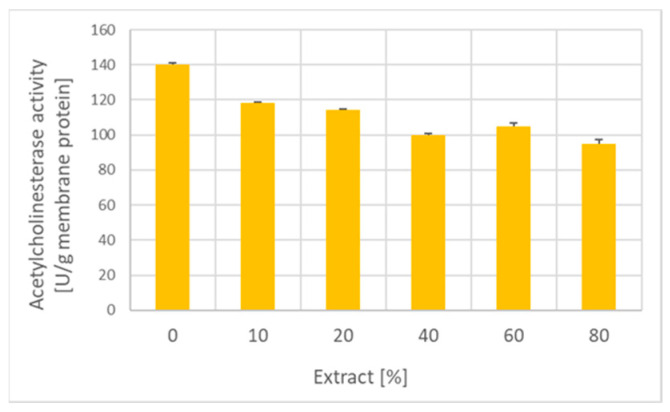
Effect of incubation with the garlic extract on the acetylcholinesterase activity of erythrocyte membranes.

The garlic extract concentration-dependently inhibited the activity of erythrocyte membrane acetylcholinesterase (Figure 10).

## 3. Discussion

Garlic extract contains allicin and other OSCs, which penetrate the cell membrane [29] and react with membrane and intracellular thiols. HPLC analysis of the extract used by us revealed the presence of *S*-ethylcysteine sulfoxide (2.31 ± 0. 10 μg/mL), γ-glutamylallylcysteine (17.97 ± 0.99 μg/mL), γ-glutamylpropenylcysteine (5.25 ± 0.17 μg/mL), γ-glutamylallylthiocysteine (5.59 ± 0.25 μg/mL), allicin (40.43 ± 2.59 μg/mL), and S-allylcysteine (7.40 ± 0.20 μg/mL) [28]. The present study revealed depletion of membrane and intracellular thiols, especially glutathione. OSCs can be considered as a general sulfhydryl reagent, resulting in the formation of *S*-allyl derivatives [30,31]. The main product of the reaction of glutathione with allicin is *S*-allylmercaptoglutathione [29]. In erythrocytes, garlic extract was reported to modify the intraerythrocytic hemoglobin, resulting in the formation of allyl disulfide derivatives of beta-chain in the β-93C or β-112C residue [32]. Several compounds able to oxidize hemoglobin and other erythrocyte constituents were isolated from the garlic, including  bis-2-propenyl trisulfide, bis-2-propenyl tetrasulfide, bis-2-propenyl pentasulfide, *bis*-2-propenyl thiosulfonate, 2-propene-1-sulfinothioic acid *S*-methyl ester, 2-propene-1-sulfinothioic acid *S*-(E)-1-propenyl ester, trans-sulfurous acid allyl ester, and 3-allylsulfanyl-allyl ester [33]. The loss of GSH and GSSG observed in this study was apparently due to the formation of OSC–GSH adducts. The remaining GSH was still in excess over the remaining GSSG, but the GSH/GSSG ratio decreased in garlic-treated erythrocytes, indicating a prooxidative shift in the erythrocyte redox environment [34].

Incubation with the garlic extract induced the binding of hemoglobin to the erythrocyte membrane. This binding seems to involve a non-covalent but also a covalent component as demonstrated by the appearance of the hemoglobin band in erythrocyte membranes separated by SDS-PAGE under reducing conditions. Apparently, this binding of hemoglobin to the membrane is one symptom of aggregation of oxidized and denatured hemoglobin, which also results in Heinz body formation, also observed in erythrocytes treated with the garlic extract.

It was reported that allicin can inactivate thiol-dependent enzymes; in some cases, this inactivation could be reversed by glutathione, while in other cases it was irreversible [29]. It can also be expected that, in other cells, thiol depletion and inactivation of thiol-dependent proteins may be the first effects of exposure to garlic extract, more so if they lack hemoglobin, which is an important sink for OSCs due to its high concentration in erythrocytes. These effects may be at least partly reversible, since depletion of cellular glutathione results in the activation of the Nrf2 signaling pathway, leading to the synthesis of glutathione-producing enzymes and restoration of glutathione levels [35]. Another effect of exposure to garlic extract is the oxidation of hemoglobin to methemoglobin and the formation of Heinz bodies. Although garlic ingestion cannot be expected to induce significant methemoglobinemia at the whole-body level due to the high amount of circulating erythrocytes, Heinz body formation was reported in some cases, as mentioned in the Introduction, apparently depending on the overall redox status of the body.

Incubation with garlic extract induced slight hemolysis, a phenomenon that could have easily been overlooked in a previous study [29]. Prolonged incubation of erythrocytes at 4 °C revealed a stronger induction of delayed hemolysis, due to changes in membrane structure and permeability. One such change concerns the permeability of the erythrocyte membrane for chloride. As demonstrated by Aubert and Motais [36], the time of hemolysis of erythrocytes in isotonic solutions of ammonium salts is a measure of permeability of the membrane for the anion component of the salt, dependent on the erythrocyte anion exchanger (Band 3 protein). Slowing down of hemolysis in isotonic NH_4_Cl solution by incubation with the garlic extract indicates inhibition of the Band 3 function, most probably due to thiol modification. Although Band 3 has no cysteine residues that are necessary for transport, it is known that treatment with the thiol-reactive *N*-ethylmaleimide or oxidative stress inhibits Band-3-mediated anion transport [37]; garlic OSCs apparently invoke the same effect.

In this study, an increase in erythrocyte membrane fluidity by garlic extract was found by measurements of both pyrene excimer fluorescence and EPR spectra of spin-labeled steric acid. This finding complements the literature data on the effects of garlic components on membrane fluidity. Garlic acid OSCs, especially diallyl trisulfide and diallyl disulfide (but not alliin), were found to increase the rigidity of model phospholipid membranes containing unsaturated phospholipids and cellular membranes at concentrations of 20–500 μM. It was suggested that membrane rigidification may contribute to the antiproliferative effects of garlic on tumor cells. However, garlic OSCs decreased the rigidity of DPPC and DPPC: cholesterol membranes [38]. In turn, alliin exerted a disordering effect on DMPC vesicles [39]. It should be taken into account that, apart from OSCs, garlic extract contains a variety of components that may interact with erythrocyte membranes, so the final effect is hard to predict based on studies of selected compounds.

The loss of EPR signal of the lipophilic spin probes, 5-NS and 16-NS, induced by the garlic extract is noteworthy. While water-soluble nitroxides are easily reduced by ascorbate, lipid-soluble nitroxide probes are resistant to such reduction [40]. Such a phenomenon was reported in erythrocytes infected with the malarial parasite *Plasmodium berghei* [41]. The reduction in EPR signal of the fatty acid spin probes observed in this study was apparently caused by lipid-soluble OSCs accumulating in the membrane.

The present study mainly demonstrates the prooxidant action of garlic extract on erythrocytes. Apart from OSCs, garlic extract also contains a plethora of antioxidants including, i.a., polyphenols and ascorbate, which can protect erythrocytes against exogenous and endogenous oxidants [8,42]. OSCs themselves also have antioxidant properties, trapping free radicals [43,44]. Indeed, rat erythrocytes were reported to be dose-dependently protected against *t*-butyl-hydroperoxide-induced lipid peroxidation, and loss of deformability, ATP, and 2,3-DPG by aged garlic extract present during the exposure to the oxidant [45]. Aged garlic extracts inhibited the formation of dense sickle cells in vitro [46]. However, under conditions of exposure of erythrocytes to the garlic extract in the absence of external oxidants, the prooxidant effects definitely predominated.

This study showed the inhibition of erythrocyte acetylcholinesterase by garlic extract. Inhibition of acetylcholinesterase by allicin [47] and essential garlic oils [48] was reported previously and speculated to have the potential to improve learning and memory and ameliorate the decline of cognitive function and memory loss associated with Alzheimer’s disease [47,49]. This aspect does not concern the erythrocyte acetylcholinesterase, whose function is still a mystery, but the decrease in the activity of this enzyme may be a marker of exposure to garlic.

## 4. Materials and Methods

### 4.1. Reagents and Equipment

2,2′-Azino-bis (3-ethylobenzthiazoline-6-sulfonic acid) (ABTS; CAS no. 504-14-6; cat. no. 10102946001; purity ≥ 99%) was provided by Roche (Warsaw, Poland). Iron(III) chloride (FeCl_3_) (CAS no. 7705-08-0; cat. no. 451649; purity ≥ 99.99%) was obtained from MedChemExpress (Monmouth Junction, NJ, USA). 2,4,6-Tris(2-pyridyl)-s-triazine (TPTZ; CAS no. 3682-35-7; cat. no. T1253; purity ≥ 98%), TEMPOL (4-hydroxy TEMPO) (CAS no. 2226-96-2; cat. no. 176141), 2′,7′-dichlorodihydrofluorescein diacetate (H_2_DCFDA) (CAS no. 2044-85-1; cat. no. 35845; purity ≥ 95%), N-ethylmaleimide (NEM; CAS no.128-53-0; cat. no. E3876), trichloroacetic acid (TCA; CAS no. 76-03-9; cat. no. T4885), L-ascorbic acid (CAS no. 50-81-7; cat. no. A0278), *ortho*-phtaldialdehyde (OPA; CAS no. 643-79-8; cat. no. P1378), sodium dodecyl sulfate (SDS) (CAS no.151-21-3; cat. no. L4509, purity ≥ 98.5%), Diethylenetriaminepentaacetic acid (DTPA) (CAS no. 67-43-6; cat. no. D1133), and dimethyl sulfoxide (DMSO) (CAS no. 67-68-5; cat. no. D2438) were provided by Merck (Poznan, Poland). Lipid Peroxidation Sensor (4,4-difluoro-5-(4-phenyl-1,3-butadienyl)-4-bora-3a,4a-diaza-s-indacene-3-un-decanoic acid; C11-BODIPY^®^ C11 (CAS no. 217075-36-0, cat. no. D3861) was purchased from Thermo Fisher Scientific (Waltham, MA, USA). Phosphate-buffered saline (PBS; cat. no. PBS404.200), sodium phosphate monobasic (CAS no. 10049-21-5; cat. no. SPM306.500, purity 98–103%), and sodium phosphate dibasic (CAS no. 7782-85-6; cat. no. SPD579.1, purity 98–102%) produced by BioShop Canada Inc. (Burlington, ON, Canada) were purchased from Lab Empire (Rzeszow, Poland). Ammonium chloride (CAS no. 12125-02-9; cat. no. 111372607; purity ≥ 99.5%), urea (CAS no. 57-13-6; cat. no. 116615309; purity ≥ 99.5%), Tris-HCl (CAS no. 77-86-1; cat. no. 118534707, purity ≥ 99%), ammonium sulfate (CAS no. 7783-20-2; cat. no. 7783-20-2; purity ≥ 98.5%), and sodium chloride (NaCl) (CAS no. 7647-14-5; cat. no. 363-117941206; purity ≥ 99.9% were provided by Chempur (Piekary Śląskie, Poland). Ammonium acetate (CAS no. 631-61-8; cat. no. 102611552; purity ≥ 98%), Triton X-100 (CAS no. 9036-19-5; cat. no. 9002-93-1), DTNB (Ellman’s Reagent) (5,5-dithio-*bis*-(2-nitrobenzoic acid) 5,5′-dithiobis-(2-nitrobenzoic acid) (CAS no. 69-78-3; cat. no. D8130), pyrene (CAS no. 129-00-0; cat. no. 2648; purity ≥ 98%), potassium hexacyanoferrate(III) (K_3_[Fe(CN)_6_]) (CAS no. 13746-66-2; cat no. 237-323-3), acetylcholine iodide (CAS no. 2260-50-6; cat. no. A7000; purity ≥ 97%), sodium pyruvate (CAS no. 504-17-6; cat. no. P2256; purity ≥ 99%), spin probes 5-doxyl stearic acid (5DS) (CAS no. 29545-48-0; cat. no. 253634), 16-doxyl stearic acid (16DS) (CAS no. 53034-38-1; cat. no. 253596), 2,2′-Azobis(2-methylpropionamidine) dihydrochloride (AAPH) (CAS no. 2997-92-4; cat. no. 440914; purity ≥ 97%), β-Nicotinamide adenine dinucleotide (NADH) (CAS no. 606-68-8; cat. no. N8129; purity ≥ 97%), and glutathione (GSH) (CAS no. 70-18-8; cat. no. Y0000517) were provided by Sigma-Aldrich (St. Louis, MO, USA). Albumin Fraction V (BSA) (CAS no. 9038-46-8; cat. no. A139, purity ≥ 97%) was bought from AppliChem (Darmstadt, Germany). 2-Thiobarbituric acid (TBA) (CAS no. 504-17-6; cat. no. 36108; purity ≥ 99%) was purchased from Serva Electrophoresis GmbH (Heidelberg, Germany). TBA was dissolved in 0.1 M NaOH at a concentration of 0.67%. Spin probes [5-doxyl stearic acid (5-DS) and 16-doxyl stearic acid (16-DS)] and all other reagents, if not stated otherwise, were purchased from Sigma-Alrich (Poznan, Poland) and were of analytical grade.

Distilled water was purified using a Milli-Q system (Millipore, Bedford, MA, USA). Transparent flat-bottom 96-well plates (cat. no. 655101) as well as black-flat-bottom 96-well plates 655209) (Greiner, Kremsmünster, Austria) were used for the assays. Fluorometric and absorptiometric measurements were performed in a Spark multimode microplate reader (Tecan Group Ltd., Männedorf, Switzerland). Images were captured with the OLYMPUS BX-51 epifluorescence microscope equipped with a DP-72 digital camera and cellSens Dimension v1.0 software. Human erythrocytes were also analyzed using an Olympus CKX53 microscope with a U-TV0.5XC-3 digital microscope camera (Olympus, Warsaw, Poland).

Electron paramagnetic resonance (EPR) measurements were performed on a Bruker multifrequency and multi-resonance FT-EPR ELEXSYS E580 spectrometer (Bruker Analytische Messtechnik, Rheinstetten, Germany) operating at the X-band (9.378989 GHz). All measurements were performed in triplicate and repeated at least three times.

### 4.2. Ethical Approval of Experiments with Blood Plasma

This study was approved by the Bioethics Committee of the University of Lodz (Permit No. 9/KBBN-UŁ/I/2020).

### 4.3. Erythrocytes and Erythrocyte Membranes

Blood was taken from a healthy volunteer (a 46-year-old woman) and anticoagulated with citrate. Erythrocytes were obtained by centrifugation and washing (3 times) with phosphate-buffered saline (PBS). Erythrocyte membranes were prepared from erythrocytes obtained from a local blood bank in Łódź, according to the method of Dodge et al. [50] with a small modification [51]. Preparations obtained from different donors were pooled.

### 4.4. Preparation of the Garlic Extract

Fresh garlic (*Allium sativum* L.) bulbs grown in Spain were purchased in a local grocery shop. A portion of cut garlic cloves (about 5 g) homogenized with 9-volume portions of PBS. The prepared samples were mixed for 10 min on ice, frozen, thawed, and centrifuged at 4000× *g* for 15 min. The supernatants obtained by centrifugation were pooled, aliquoted into 2 mL Eppendorf tubes and used immediately or frozen at −80 °C and used after thawing. Unless stated otherwise, 10% erythrocyte suspension in PBS or erythrocyte membranes (3 mg protein/mL) were exposed to the extract diluted with PBS at various proportions, at 37 °C, with shaking, for 1 h. Then the erythrocytes or membranes were washed three times with PBS and analyzed.

### 4.5. Observation of Heinz Bodies

Erythrocytes were stained supravitally with methyl violet and observed using the OLYMPUS BX-51 epifluorescence microscope equipped with a DP-72 digital camera and cellSens Dimension v1.0 software. The presence of Heinz bodies was confirmed turbidimetrically by a modified method of Winterbourn [52]. Briefly, hemolysates were obtained by lysis of 20 μL of incubated erythrocytes with 500 μL of deionized water and OD of a 200 μL aliquots was measured at 700 nm in wells of a 96-well plate, using a microplate reader, without centrifugation and after centrifugation (12,000× *g*, 5 min).

### 4.6. Hemoglobin Oxidation

Erythrocytes incubated with the garlic extract and washed were hemolyzed with deionized water and centrifuged. Absorption spectra of the supernatants were measured. As a measure of the extent of hemoglobin oxidation to methemoglobin, the ratio of absorbance at 630 nm to that at 577 nm was used.

### 4.7. Hemoglobin Binding to the Membrane

Erythrocytes were isolated with various dilutions of the garlic extract and after washing off the extract, erythrocyte membranes were prepared. Hemoglobin content in membranes was estimated by dissolving the membranes in 1% SDS and measurement of absorbance at the maximum of the Soret band of SDS-denatured hemoglobin found to correspond to the wavelength of 410 nm. The obtained membranes were also subject to SDS electrophoresis in 12% acrylamide gels under reducing conditions, stained with Coomassie Brilliant Blue R, and density of protein bands was quantified with ImageJ software Version 1.51w (NIH, Bethesda, MD, USA, http://rsb.info.nih.gov/ij/, accessed 23 December 2023).

### 4.8. Estimation of the Extent of Hemolysis

After incubation with the extract, the erythrocytes were sedimented and hemoglobin concentration was measured in the supernatants. As oxidation of hemoglobin took place in the presence of the extract, absorbance was measured at 527 nm, an isosbestic point in the hemoglobin/methemoglobin system.

### 4.9. Measurement of Hemolysis in Isotonic Ammonium Chloride

Suspension of erythrocytes incubated with the extract (20 μL) was added to 200 μL of isotonic (150 mM) ammonium chloride and, after thorough mixing, the decrease in optical density of the samples was followed at 700 nm to determine the time of decrease to 0.5 of the initial value.

### 4.10. Estimation of the Acid-Soluble Thiol Level

Erythrocyte suspensions (200 μL) were added with 25 μL of 20% trichloroacetic acid (TCA), incubated on ice for 10 min, and centrifuged, and 50 μL of the supernatant was added to a microplate well containing 200 μL of 0.2 M phosphate buffer, pH 7.6. Then, 3 μL of 10 mM solution of 5,5-dithio-bis-(2-nitrobenzoic acid) (DTNB) was added, and after 15 min, absorbance was measured at 412 nm. Thiol concentration was calculated using the molar absorption coefficient of the thionitrobenzoate anion of 14.15 × 10^3^ M^−1^ cm^−1^ [53].

### 4.11. Estimation of Thiol Groups Content of the Membranes

Aliquots (200 μL) of erythrocyte membranes (protein concentration: 1.5 mg/mL) in 0.1 M phosphate buffer, pH 7.4 were added with 20 μL of 5% sodium dodecyl sulfate (SDS) and 3 μL of 10 mM DTNB. After 15 min incubation in the dark, absorbance was measured at 412 nm.

### 4.12. Estimation of the Glutathione Level

The concentration of GSH and GSSG was determined fluorimetrically with *o*-phtaldehyde [54], as described previously [35].

### 4.13. Estimation of the Redox Potential of the Erythrocyte Glutathione System

The redox potential of the erythrocyte 2 GSH/GSSG system was calculated according to Schafer and Buettner [34]:

E_h_ = −264 − (59.1/2) log([GSH]^2^/[GSSG]) mV,

assuming intracellular pH to be 7.4 and not to change in the presence of the garlic extract.

### 4.14. Estimation of the Level of Intracellular Reactive Oxygen Species

Samples containing 50 μL of 10% suspensions were added with 100 μL of PBS or various dilutions of garlic extract with PBS and incubated with shaking for 45 min. Then, 1 μL of 1 mM solution of H_2_DCF-DA was added and incubation was continued for the next 15 min. Afterward, fluorescence was measured at 485/538 nm.

### 4.15. Estimation of Lipid Peroxidation

BODIPY^®^ 581/591 undecanoic acid (BODIPY C11) was dissolved in DMSO by adding 475 μL DMSO to 25 μL 2 mM BODIPY C11 stock solution (dissolved also DMSO). Erythrocyte membranes were added with PBS or various dilutions of the garlic extract (final concentration: 600 μg protein/mL) and BODIPY C11 to the final concentration of 2.5 μM and incubated at 37 °C. Then the fluorescence spectra of the samples in the range of 500–650 nm were measured using the excitation wavelength of 460 nm. The ratio of fluorescence at 526 nm and 598 nm was taken as a measure of lipid peroxidation.

Alternatively, lipid peroxidation was assessed with thiobarbituric acid. In brief, membrane sediment containing 150 μg protein were added with 125 μL of 10% trichloroacetic acid and 125 μL of 0.67% thiobarbituric acid solution in 0.1 M NaOH, mixed, and incubated at 100 °C for 20 min. Then the samples were cooled, centrifuged, and absorbance of 200 μL of the supernatants was measured at 532 nm in a plate reader.

### 4.16. Carbonylation of Membrane Proteins

Membrane protein carbonyls were assessed with 2,4-dinitrophenylhydrazine according to Levine et al. [55].

### 4.17. Estimation of Membrane Fluidity with Pyrene

Erythrocyte membranes incubated with PBS or various concentrations of the extract were added with pyrene (final concentration of 17 μM) from a concentrate stock solution in DMSO. The final membrane concentration corresponded to 200 μg protein/mL. After 15 min incubation, fluorescence spectra were taken in the range of 370–500 nm (excitation: 330 nm). The ratio of the excimer fluorescence peak (466 nm) to that of the monomer fluorescence peak (386 nm) was taken as a measure of membrane fluidity.

### 4.18. Estimation of Membrane Fluidity with Spin Probes

Erythrocyte membranes (3 mg protein/mL) were added with 10 mM spin probes (5-DS or 16-DS) to a final concentration of 10 μM and incubated with various dilutions of the garlic extract. Then, the extract was washed off, and electron paramagnetic resonance (EPR) measurements of the spin-labeled membranes were performed using microhematocrit capillaries (non-heparinized microhematocrit tubes ~75 μL; 1.55 mm × 75 mm; Medlab Products, Raszyn, Poland) in a BRUKER multifrequency and multi-resonance FT-EPR ELEXSYS E580 CW-EPR spectrometer (BRUKER BIOSPIN, Billerica, MA, USA) [X-band (~9.5 GHz) with an ER4119-HS cavity]. Sample capillaries were placed into a quartz EPR sample tube and centered in a microwave cavity. The following settings were used: central field, 3353 G; modulation amplitude, 1 G; modulation frequency, 100 kHz; microwave power, 23.77 mW; power attenuation 2 dB; scan range, 100 G; conversion time, 25 ms; sweep time, 25.6 s. The spectra were recorded in 1024 channels, and the number of accumulated scans was 3. The EPR spectra were recorded and analyzed using Xepr 2.6b.74 software.

Rotational correlation time τ_c_ was calculated using the following equation:

$$\tau_{c}=6.5\times 10^{-10\;\;}W_{0\;}(\sqrt{\frac{h_{0}}{h_{-1}}}-1)$$

where τ_c_—time when the spin probe undergoes full rotation; *W*_0_—width of the mid-line spectrum (in G); *h*_0_—the height of the mid-line spectrum; *h*_−1_—the height of the high-field line of the spectrum [56].

Order parameter (*S*) was calculated according to the following formula:

$$S=\frac{2A_{∥}-2A_{⊥}}{2\;[A_{zz}-\frac{A_{xx}+A_{yy}}{2}]}$$

taken from Ref. [57], where 2*A*_∥_ and 2*A*_┴_ are the separations between the outer and inner extrema, respectively, in the experimental spectrum, and *A_xx_*, *A_yy_*, and *A_zz_* are the values of the principal components of the hyperfine tensor (*A_xx_* = *A_yy_* = 6 G, *A_zz_* = 32 G) [58].

### 4.19. Determination of Acetylcholinesterase Activity

Acetylcholinesterase activity of erythrocyte membranes was assayed by a slight modification of the method of Ellman [59]. Erythrocyte membranes (10 μL; protein concentration of 300 μg/mL) were added to the assay medium containing 500 μM acetylthiocholine iodide and 500 μM DTNB in 100 mM sodium phosphate buffer, pH 7.4. The increase in absorbance was monitored at 412 nm for 2 min and the rate of absorbance increase was taken as a measure of the enzyme activity, using the molar absorption coefficient of the thionitrobenzoate anion of 14.15 × 10^3^ M^−1^ cm^−1^ [53].

### 4.20. Statistical Analysis

The results are presented as means ± SD from at least three independent experiments. To estimate the statistical significance of differences, Student’s *t*-test was employed. *p* < 0.05 was considered statistically significant. Statistical analysis of the data was performed using the STATISTICA software package (version 13.1, Statsoft Inc. 2016, Tulsa, OK, USA).

## Figures and Tables

**Table 1:** Effect of garlic extract on the concentration of reduced (GSH) and oxidized glutathione (GSSG) in erythrocytes.

Extract Concentration [%]	GSH [μM]	GSSG [μM]	GSH/GSSG	E_h_ of the GSH/GSSG Redox Couple [mV]
0	1880.7 ± 53.3	112.5 ± 14.5	16.7	−220
2.5	68.4 ± 3.2	20.7 ± 2.2	3.3	−156
5	31.6 ± 5.2	19.7 ± 2.3	1.6	−137
10	21.7 ± 2.9	16.5 ± 3.2	1.3	−130
20	19.3 ± 4.1	10.7 ± 2.8	1.8	−132
50	17.8 ± 1.8	9.7 ± 1.5	1.8	−131

**Table 2:** Effect of incubation with garlic extract on the binding of hemoglobin to the membrane estimated from absorbance at the Soret band and electrophoresis of membrane proteins (reducing conditions).

Garlic Extract [%]	Absorbance at 410 nm	Hemoglobin as % of Total Membrane Protein
0	0.075 ± 0.011	0.5 ± 0.3
12.5	0.663 ± 0.042	5.9 ± 0.9
25	0.769 ± 0.038	6.6 ± 0.8
37.5	0.834 ± 0.045	8.5 ± 1.1
50	0.880 ± 0.061	10.1 ± 1.7

**Table 3:** Effect of garlic extract on the fluorescence ratio of BODIPY C11 probe after 1 h incubation (37 °C) in the absence and in the presence of erythrocyte membranes. Membranes (200 μg protein/mL) or 5 mM phosphate buffer, pH 7.4, constituted 50% of the volume of the reaction medium.

Extract Concentration [%]	F(526 nm)/F(598 nm)	
	Without Erythrocyte Membranes	With Erythrocyte Membranes
0	0.062 ± 0.002	0.085 ± 0.009
5	0.121 ± 0.005	0.102 ± 0.011
12.5	0.130 ± 0.007	0.131 ± 0.017
25	0.140 ± 0.005	0.139 ± 0.009
50	0.211 ± 0.012	0.330 ± 0.012

**Table 4:** Effect of incubation with the garlic extract on the membrane fluidity estimated by pyrene fluorescence.

Extract Concentration [%]	Pyrene Excimer to Monomer Fluorescence Ratio
0	0.411 ± 0.004
5	0.469 ± 0.006
12.5	0.453 ± 0.009
25	0.462 ± 0.004
50	0.436 ± 0.003

**Table 5:** Effect of incubation with the garlic extract on the membrane fluidity estimated with 5-DS and 16-DS spin probes.

Extract Concentration [%]	Order Parameter S of 5-DS	Rotational Correlation Time τ_c_ of 16-DS [ns]
0	0.640 ± 0.009	1.56 ± 0.25
10	0.632 ± 0.017	1.37 ± 0.15
25	0.628 ± 0.05	1.24 ± 0.04

## Data Availability

Data will be available from the corresponding author upon reasonable request.

## References

[1] Ansary J., Forbes-Hernández T.Y., Gil E., Cianciosi D., Zhang J., Elexpuru-Zabaleta M., Simal-Gandara J., Giampieri F., Battino M. (2020). Potential health benefit of garlic based on human intervention studies: A brief overview. Antioxidants.

[2] Espinoza T., Valencia E., Albarrán M., Díaz D., Quevedo R.A., Díaz O., Bastías J. (2020). Garlic (*Allium sativum* L.) and its beneficial properties for health: A review. Agroind. Sci..

[3] Netzel M.E. (2020). Garlic: Much more than a common spice. Foods.

[4] Bhatwalkar S.B., Mondal R., Krishna S.B.N., Adam J.K., Govender P., Anupam R. (2021). Antibacterial properties of organosulfur compounds of garlic (*Allium sativum*). Front. Microbiol..

[5] Rouf R., Uddin S.J., Sarker D.K., Islam M.T., Ali E.S., Shilpi J.A., Nahar L., Tiralongo E., Sarker S.D. (2020). Antiviral potential of garlic (*Allium sativum*) and its organosulfur compounds: A systematic update of pre-clinical and clinical data. Trends Food Sci. Technol..

[6] Pandey P., Khan F., Alshammari N., Saeed A., Aqil F., Saeed M. (2023). Updates on the anticancer potential of garlic organosulfur compounds and their nanoformulations: Plant therapeutics in cancer management. Front. Pharmacol..

[7] Ruhee R.T., Roberts L.A., Ma S., Suzuki K. (2020). Organosulfur compounds: A review of their anti-inflammatory effects in human health. Front. Nutr..

[8] Bozin B., Mimica-Dukic N., Samojlik I., Goran A., Igic R. (2008). Phenolics as antioxidants in garlic (*Allium sativum* L., Alliaceae). Food Chem..

[9] Škrovánková S., Mlček J., Snopek L., Planetová T. (2018). Polyphenols and antioxidant capacity in different types of garlic. Potravin. Slovak J. Food Sci..

[10] Kopec A., Piatkowska E., Leszczynska T., Sikora E. (2013). Healthy properties of garlic. Curr. Nutr. Food Sci..

[11] Azzini E., Durazzo A., Foddai M.S., Temperini O., Venneria E., Valentini S., Maiani G. (2014). Phytochemicals content in Italian garlic bulb (*Allium sativum* L.) varieties. J. Food Res..

[12] Borlinghaus J., Albrecht F., Gruhlke M.C., Nwachukwu I.D., Slusarenko A.J. (2014). Allicin: Chemistry and biological properties. Molecules.

[13] DeLeon E.R., Gao Y., Huang E., Olson K.R. (2016). Garlic oil polysulfides: H_2_S-and O_2_-independent prooxidants in buffer and antioxidants in cells. Am. J. Physiol. Reg. Integr. Comp. Physiol..

[14] Kulikova V.V., Morozova E.A., Koval V.S., Solyev P.N., Demidkina T.V., Revtovich S.V. (2023). Thiosulfinates: Cytotoxic and antitumor activity. Biochemistry (Mosc.).

[15] Hamlaoui S., Mokni M., Limam N., Zouaoui K., Ben Rayana M.C., Carrier A., Limam F., Amri M., Marzouki L., Aouani E. (2012). Protective effect of grape seed and skin extract on garlic-induced erythrocyte oxidative stress. J. Physiol. Pharmacol..

[16] Salami H.A., Tukur M.A., Bukar A., John I.A., Abubakar A., Jibrin J. (2018). High reticulocyte count with abnormal red blood cell morphology in normal Wistar rats after garlic administration. Niger. J. Physiol. Sci..

[17] Oboh G. (2004). Prevention of garlic-induced hemolytic anemia using some tropical green leafy vegetables. J. Med. Food.

[18] Hamlaoui-Gasmi S., Mokni M., Aouani E., Amri M., Marzouki L. (2011). Modulation of hematological parameters by garlic based on route of administration in rat. J. Food Biochem..

[19] Lee K.W., Yamato O., Tajima M., Kuraoka M., Omae S., Maede Y. (2000). Hematologic changes associated with the appearance of eccentrocytes after intragastric administration of garlic extract to dogs. Am. J. Vet. Res..

[20] Kempaiah R.K., Srinivasan K. (2004). Influence of dietary curcumin, capsaicin and garlic on the antioxidant status of red blood cells and the liver in high-fat-fed rats. Ann. Nutr. Metab..

[21] Kempaiah R.K., Srinivasan K. (2006). Beneficial influence of dietary curcumin, capsaicin and garlic on erythrocyte integrity in high-fat fed rats. J. Nutr. Biochem..

[22] Zalejska-Fiolka J., Kasperczyk A., Kasperczyk S., Błaszczyk U., Birkner E. (2006). Effect of garlic supplementation on erythrocytes antioxidant parameters, lipid peroxidation, and atherosclerotic plaque formation process in oxidized oil-fed rabbits. Biol. Trace Elem. Res..

[23] Sarkar A., Sengupta D., Mandal S., Sen G., Dutta Chowdhury K., Chandra Sadhukhan G. (2015). Treatment with garlic restores membrane thiol content and ameliorates lead induced early death of erythrocytes in mice. Env. Toxicol..

[24] Tikare S.N., Saeed Y., Amrita D.G., Salim A.D., Kusal K.D. (2012). Effect of garlic (*Allium sativum*) on hematology and erythrocyte antioxidant defense system of albino rats exposed to heavy metals (nickel ii & chromium vi). J. Physiol. Pharmacol..

[25] Avcı A., Atlı T., Ergüder İ.B., Varlı M., Devrim E., Aras S., Durak İ. (2008). Effects of garlic consumption on plasma and erythrocyte antioxidant parameters in elderly subjects. Gerontology.

[26] Takasu J., Uykimpang R., Sunga M.A., Amagase H., Niihara Y. (2006). Aged garlic extract is a potential therapy for sickle-cell anemia. J. Nutr..

[27] Salami H.A., John A.I., Ekanem A.U. (2012). The effect of aqueous preparation of *Allium Cepa* (onion) and *Allium Sativa* (garlic) on erythrocyte osmotic fragility in Wistar rats: In vivo and in vitro studies. Niger. J. Physiol. Sci..

[28] Furdak P., Pieńkowska N., Kapusta I., Bartosz G., Sadowska-Bartosz I. (2023). Comparison of antioxidant and antiproliferative effects of various forms of garlic and ramsons. Molecules.

[29] Miron T., Rabinkov A., Mirelman D., Wilchek M., Weiner L. (2000). The mode of action of allicin: Its ready permeability through phospholipid membranes may contribute to its biological activity. Biochim. Biophys. Acta Biomembranes.

[30] Rabinkov A., Miron T., Konstantinovski L., Wilchek M., Mirelman D., Weiner L. (1998). The mode of action of allicin: Trapping of radicals and interaction with thiol containing proteins. Biochim. Biophys. Acta Gen. Sub..

[31] Miron T., Shin I., Feigenblat G., Weiner L., Mirelman D., Wilchek M., Rabinkov A. (2002). A spectrophotometric assay for allicin, alliin, and alliinase (alliin lyase) with a chromogenic thiol: Reaction of 4-mercaptopyridine with thiosulfinates. Anal. Biochem..

[32] Bonaventura J., Rodriguez E.N., Beyley V., Vega I.E. (2010). Allylation of intraerythrocytic hemoglobin by raw garlic extracts. J. Med. Food.

[33] Hu Q., Yang Q., Yamato O., Yamasaki M., Maede Y., Yoshihara T. (2002). Isolation and identification of organosulfur compounds oxidizing canine erythrocytes from garlic (*Allium sativum*). J. Agric. Food Chem..

[34] Schafer F.Q., Buettner G.R. (2001). Redox environment of the cell as viewed through the redox state of the glutathione disulfide/glutathione couple. Free Radic. Biol. Med..

[35] Pieńkowska N., Bartosz G., Sadowska-Bartosz I. (2023). Effect of 6-hydroxydopamine increase the glutathione level in SH-SY5Y human neuroblastoma cells. Acta Biochim. Pol..

[36] Aubert L., Motais R. (1975). Molecular features of organic anion permeability in ox red blood cell. J. Physiol..

[37] Jennings M.L. (2021). Cell physiology and molecular mechanism of anion transport by erythrocyte band 3/AE1. Am. J. Physiol.-Cell Physiol..

[38] Tsuchiya H., Nagayama M. (2008). Garlic allyl derivatives interact with membrane lipids to modify the membrane fluidity. J. Biomed. Sci..

[39] Ezer N., Sahin I., Kazanci N. (2017). Alliin interacts with DMPC model membranes to modify the membrane dynamics: FTIR and DSC Studies. Vibrat. Spectrosc..

[40] Sadowska-Bartosz I., Bartosz G. (2024). The cellular and organismal effects of nitroxides and nitroxide-containing nanoparticles. Int. J. Mol. Sci..

[41] Deslauriers R., Butler K., Smith I.C. (1987). Oxidant stress in malaria as probed by stable nitroxide radicals in erythrocytes infected with *Plasmodium berghei*. The effects of primaquine and chloroquine. Biochim. Biophys. Acta Mol. Cell Res..

[42] Banerjee S.K., Mukherjee P.K., Maulik S.K. (2003). Garlic as an antioxidant: The good, the bad and the ugly. Phytother. Res..

[43] Vaidya V., Ingold K.U., Pratt D.A. (2009). Garlic: Source of the ultimate antioxidants–sulfenic acids. Angew. Chem. Int. Ed. Engl..

[44] Amorati R., Lynett P.T., Valgimigli L., Pratt D.A. (2012). The reaction of sulfenic acids with peroxyl radicals: Insights into the radical-trapping antioxidant activity of plant-derived thiosulfinates. Chemistry.

[45] Moriguchi T., Takasugi N., Itakura Y. (2001). The effects of aged garlic extract on lipid peroxidation and the deformability of erythrocytes. J. Nutr..

[46] Ohnishi S.T., Ohnishi T. (2001). In vitro effects of aged garlic extract and other nutritional supplements on sickle erythrocytes. J. Nutr..

[47] Kumar S. (2015). Dual inhibition of acetylcholinesterase and butyrylcholinesterase enzymes by allicin. Indian J. Pharmacol..

[48] Akinyemi A.J., Faboya L., Awonegan A., Olayide I., Anadozie S., Oluwasola T. (2018). Antioxidant and anti-acetylcholinesterase activities of essential oils from garlic (*Allium sativum*) bulbs. Int. J. Plant Res..

[49] Mukherjee D., Banerjee S. (2013). Learning and memory promoting effects of crude garlic extract. Indian J. Exp. Biol..

[50] Dodge J.T., Mitchell C., Hanahan D.J. (1963). The preparation and chemical characteristics of hemoglobin-free ghosts of human erythrocytes. Arch. Biochem. Biophys..

[51] Soszynski M., Filipiak A., Bartosz G., Gebicki J.M. (1996). Effect of amino acid peroxides on the erythrocyte. Free Radic. Biol. Med..

[52] Winterbourn C.C. (1979). Protection by ascorbate against acetylphenylhydrazine-induced Heinz body formation in glucose-6-phosphate dehydrogenase deficient erythrocytes. Br. J. Haematol..

[53] Eyer P., Worek F., Kiderlen D., Sinko G., Stuglin A., Simeon-Rudolf V., Reiner E. (2003). Molar absorption coefficients for the reduced Ellman reagent: Reassessment. Anal. Biochem..

[54] Senft A.P., Dalton T.P., Shertzer H.G. (2000). Determining glutathione and glutathione disulfide using the fluorescence probe *o*-phthalaldehyde. Anal. Biochem..

[55] Levine R.L., Garland D., Oliver C.N., Amici A., Climent I., Lenz A.G., Ahn B.W., Shaltiel S., Stadtman E.R. (1990). Determination of carbonyl content in oxidatively modified proteins. Methods Enzymol..

[56] Ježek P., Freisleben H.J. (1994). Fatty acid binding site of the mitochondrial uncoupling protein: Demonstration of its existence by EPR spectroscopy of 5-DOXYL-stearic acid. FEBS Lett..

[57] Hubbell W.L., McConnell H.M. (1971). Molecular motion in spin-labeled phospholipids and membranes. J. Am. Chem. Soc..

[58] Schreier S., Polnaszek C.F., Smith I.C. (1978). Spin labels in membranes. Problems in practice. Biochim. Biophys. Acta.

[59] Ellman G.L., Courtney K.D., Andres V., Feather-Stone R.M. (1961). A new and rapid colorimetric determination of acetylcholinesterase activity. Biochem. Pharmacol..

